# Lymphocyte Oxidative Stress/Genotoxic Effects Are Related to Serum IgG and IgA Levels in Coke Oven Workers

**DOI:** 10.1155/2014/801346

**Published:** 2014-07-20

**Authors:** Meili Gao, Yongfei Li, Aqun Zheng, Xiaochang Xue, Lan Chen, Yu Kong

**Affiliations:** ^1^Department of Biological Science and Engineering, Institute of Mitochondrial Biology and Medicine, The Key Laboratory of Biomedical Information Engineering of Ministry of Education, School of Life Science and Technology, Xi'an Jiaotong University, Xianning West Road 28, Xi'an, Shaanxi 710049, China; ^2^School of Materials and Chemical Engineering, Xi'an Technological University, Xi'an 710032, China; ^3^School of Science, Xi'an Jiaotong University, Xi'an, Shaanxi 710049, China; ^4^Department of Biopharmaceutics School of Pharmacy, State Key Laboratory of Cancer Biology, Fourth Military Medical University, Xi'an, Shaanxi 710032, China; ^5^Center of Shared Experimental Facilities, The Key Laboratory of Biomedical Information Engineering of Ministry of Education, School of Life Science and Technology, Xi'an Jiaotong University, Xi'an, Shaanxi 710049, China

## Abstract

We investigated oxidative stress/genotoxic effects levels, immunoglobulin levels, polycyclic aromatic hydrocarbons (PAHs) levels exposed in 126 coke oven workers and in 78 control subjects, and evaluated the association between oxidative stress/genotoxic effects levels and immunoglobulin levels. Significant differences were observed in biomarkers, including 1-hydroxypyrene levels, employment time, percentages of alcohol drinkers, MDA, 8-OHdG levels, CTL levels and CTM, MN, CA frequency, and IgG, IgA levels between the control and exposed groups. Slightly higher 1-OHP levels in smoking users were observed. For the dose-response relationship of IgG, IgA, IgM, and IgE by 1-OHP, each one percentage increase in urinary 1-OHP generates a 0.109%, 0.472%, 0.051%, and 0.067% decrease in control group and generates a 0.312%, 0.538%, 0.062%, and 0.071% decrease in exposed group, respectively. Except for age, alcohol and smoking status, IgM, and IgE, a significant correlation in urinary 1-OHP and other biomarkers in the total population was observed. Additionally, a significant negative correlation in genotoxic/oxidative damage biomarkers of MDA, 8-OH-dG, CTL levels, and immunoglobins of IgG and IgA levels, especially in coke oven workers, was found. These data suggest that oxidative stress/DNA damage induced by PAHs may play a role in toxic responses for PAHs in immunological functions.

## 1. Introduction

Coke oven workers are constantly exposed to coke oven emissions which are toxic chemicals, especially polycyclic aromatic hydrocarbons (PAHs). PAHs are formed during combustion of fossil fuels and typified by the 1-hydroxypyrene levels. The 1-hydroxypyrene level has been shown to be a good marker for total PAHs exposure [1, 2]. Some of PAHs are carcinogenic due to their metabolites and their ability to generate genetic damage and further oxidative DNA damage through the production of reactive oxygen species during metabolism [3, 4].

Of many indicators for oxidative DNA damage, 8-hydroxy-2′-deoxyguanosine (8-OHdG) represents an important product from oxidative damage to DNA. 8-OHdG is formed in a promutagenic DNA lesion induced by the reaction of hydroxyl radicals with guanosine at the C8 site in DNA. A growing number of surveys and occupational studies indicated that elevated levels of 8-OHdG in DNA from leukocytes or excretion in urine have been observed in PAHs exposure of smokers and workers [5–8]. Malondialdehyde (MDA), which is an end product of the oxidation of polyunsaturated fatty acids and can determine the degree of lipid peroxidation, has been used as a marker for oxidative stress [9].

It has been reported that low level PAH exposure causes DNA single strand breakage, the formation of DNA damages, and immunotoxicity [10–12]. Immunotoxicity can change lymphocytic subpopulation in peripheral blood and serum immunoglobulin levels in coke oven workers exposed to PAHs [12, 13]. As for genotoxic risk factors, the comet assay, micronucleus (MN) assay, and chromosomal aberrations (CA) assay have been used to evaluate the biomarkers of early biological effects [14]. The comet assay has been found to be a very sensitive method for measuring DNA damage. The MN test was found to provide a cytogenetic parameter and allowed the detection of both clastogenic and aneugenic agents [14]. Chromosomal damage has also been found to provide CA such as chromosome breakage, chromosome deletion, and chromosome polyploid [13, 14].

In the present study, we investigated if there was any relation between the levels of MDA, 8-OHdG, and genotoxic damages and immunoglobulin levels in serum and lymphocytes of workers exposed to coke oven emission.

## 2. Materials and Methods

### 2.1. Study Subjects

The 126 coke oven workers and 78 non-coke-oven workers who were all males and worked in the same steel company in northern China were studied in this paper. These 126 coke oven workers were in active service at the time of the study, were employed for at least 6 months, and were recruited as the exposed group. The 78 non-coke-oven workers were staff members of the offices and hospitals of the same steel company and served as the control group. The workers exposed to known mutagenic agents, such as radiotherapy and chemotherapy in the last 3 months, were excluded. Questionnaires were administered by trained interviewers to collect information on demographic information, including age, length of employment, smoking, and alcohol habits. Individuals who had smoked for 3 months were considered as smokers. Those who drank more than twice a week in the last six months were classified as drinkers. Blood samples were collected at the end of these days. In the morning, 5 mL fasting venous blood and 10 mL urine samples were collected from each subject for further analysis. The study was approved by the Ethics Committee of Xi'an Jiaotong Medical College and was performed in accordance with the Helsinki Declaration (1964).

### 2.2. 1-Hydroxypyrene in Urine Assay

Urine 1-hydroxypyrene (1-OHP) was measured by the method described by Jongeneelen and Anzion [15] and Siwińska et al. [16]. Briefly, aliquots of 10 mL urine samples were enzymatically deconjugated and transferred to primed C18 octadecyl cartridges (Beckman, USA). Then the samples were washed with 10 mL of water and eluted with 9 mL of methanol. The components of the elutae was subjected to a high pressure liquid chromatography of the Waters Alliance 2695 (Waters, USA) with the XAqua C18, 150 × 4.1 mm column. A fluorescence detector of F1000 (Hitachi, Japan) was quantitatively assayed 1-hydroxypyrene concentration. The wavelengths of excitation and emission were 229 nm and 400 nm, respectively. The concentrations of 1-OHP were normalised to urinary creatinine. Urinary creatinine concentrations were assayed by a standard colorimetric method with the picric acid reaction and absorption at 520 nm [17].

### 2.3. Serum Biomarkers Assay

Blood samples were obtained after an overnight fast. Samples (3 mL) of venous blood were incubated in a water bath for 30 minutes at 37°C and centrifuged at 4500 rpm for 10 minutes. The supernatants were stored at −70°C. The levels of serum MDA represented lipid peroxidation product were measured spectrophotometrically by a modification of the method described by Buege and Aust [18]. The spectrophotometric measurements were done with Shimadzu UV-1208 spectrophotometer (Japan). The MDA concentration was calculated using its extinction coefficient of 1.56 × 10^5^ L/mol cm at 535 nm. Enzyme linked immunosorbent assay (ELISA) was used to measure the concentrations of 8-OHdG, in accordance with the procedures described in the assay kit (Sigma, USA). Serum IgA, IgE, IgG, and IgM levels were measured by a nephelometric method [19].

### 2.4. Comet Assay, Micronuclei Test, and Chromosomal Aberrations Assay

Venous blood was collected from all subjects using heparinized syringes. Lymphocyte cultures were set up by adding 0.5 mL of heparinised blood to 4.5 mL of chromosome medium (RPMI 1640, Gibco) supplemented with 20% heat inactivated fetal bovine serum (Gibco), antibiotics (penicillin and streptomycin), and L-glutamine. Lymphocytes were stimulated by 1% phytohaemagglutinin (Gibco). The comet assay, micronuclei test, and CA assay were performed to assay the genotoxic damage of lymphocytes in workers. DNA damage to lymphocytes was assayed by the alkaline single-cell gel technique [20, 21]. Images of 25 randomly selected lymphocytes were analyzed for each sample. The slides were examined using a Comet 4.0 image analysis system fitted with an Olympus BX50 fluorescence microscope. For the collected cell sample of each subject, images of 50 randomly selected cells were analyzed for each sample. The mean level of comet tail length (CTL) and comet tail moment (CTM) of the 50 cells was as the value of the sample. The determination of micronuclei in lymphocytes was performed as described by Fenech and Morley [22]. The cultures were incubated at 37°C for 72 hours, and 44 hours after the initiation of cultures, cytochalasin-B (Sigma) at a concentration of 6 mg/mL was added to arrest cytokinesis. MN slides were stained with 10% Giemsa in phosphate buffer. A total of 1000 binucleates cells with well-preserved cytoplasm were examined by a well-trained research assistant under double blindness for each subject on coded slides throughout the study. The data are reported as the occurrence rate of ‰ micronucleated cells for each subject. The CA assay was according to the method described by Carrano and Natarajan [23]. Briefly, colcemid was added to arrest the cells in metaphase after incubating the cultures for 48 hours at 37°C; then cells were collected by centrifugation, resuspended in a prewarmed hypotonic solution (0.075 M KCl) for 25 minutes, and fixed in acetic acid : methanol (1 : 3, v/v). Air dried preparations were made and the slides were stained with Giemsa. A total of 100 well spread metaphases containing 46 ± 1 chromosomes was examined for each subject on coded slides; otherwise the data were discarded. The data are reported as the occurrence number and occurrence rate of % CA for each group.

### 2.5. Statistical Analysis

All analyses were carried out using the Statistical Package for Social Sciences (SPSS13.0). We used Mann-Whitney and Pearson chi-square tests to compare the demographics and lifestyle variables between the exposure and control groups. We used the Mann-Whitney test to compare values of biomarkers between the exposure and control groups. Spearman's rank correlation coefficient was calculated to evaluate the relations between MDA, CTL, IgG, and the impact of independent variables (occupational PAHs exposure, age, length of employment, smoking, and alcohol drinking) on dependent variables. Pearson correlation was calculated to evaluate the relations between MDA and 8-OH-dG level, 8-OH-dG and CTL level, MDA, and IgG, IgA level. All statistical tests were two-sided with a significant level of *P* < 0.05.

## 3. Results

### 3.1. Demographic Characteristics of Study Subjects

Table 1 shows the characteristics of study subjects by work site. Exposed workers were 1 year older (mean age) than control subjects. Significant differences were observed in 1-OHP levels, employment time, percentages of alcohol drinkers, MDA, 8-OHdG levels, CTL level and CTM, MN, CA frequency, IgG, and IgA between the control and exposed groups, but no significance for age, current smokers distribution, and IgM and IgE levels.

Table 2 shows the results of urinary 1-OHP relative to smoking status in exposed workers and controls, after adjustment for exposure concentration. A slight higher of 1-OHP levels in smoking users were observed both in control and exposed groups, but this was not significant (*P* = 0.142 in control and *P* = 0.096 in exposed group, resp.).

### 3.2. Dose-Response Relationships of Urinary 1-OHP with Immunoglobins and Correlation between 1-OHP and Other Biomarkers

The dose-response relationship of IgG, IgA, IgM, and IgE by 1-OHP was analyzed as in Table 3. The values of IgG, IgA, IgM, and IgE and 1-OHP were all ln-transformed in the multiple linear regression models. For the control group, each one percentage increase in urinary 1-OHP generates a 0.109% (*P* = 0.353), 0.472% (*P* < 0.001), 0.051% (*P* = 0.658), and 0.067% (*P* = 0.565) decrease in IgG, IgA, IgM, and IgE, respectively. For the exposed group, each one percentage increase in urinary 1-OHP generates a 0.312% (*P* < 0.001), 0.538% (*P* < 0.001), 0.062% (*P* = 0.592), and 0.071% (*P* = 0.420) decrease in IgG, IgA, IgM, and IgE, respectively. Correlation between 1-OHP and other indices was assayed as in Table 4. Except for age, alcohol status, current smokers, IgM, and IgE, a significant correlation in urinary 1-OHP and other biomarkers in the total population (exposed and controls) (*P* < 0.001 for other biomarkers, *P* = 0.009 for CA, resp.) was observed in the studied subjects.

### 3.3. Correlation between Oxidative/Genotoxic Damage Biomarkers and Immunoglobin Levels

**Figure 1:**
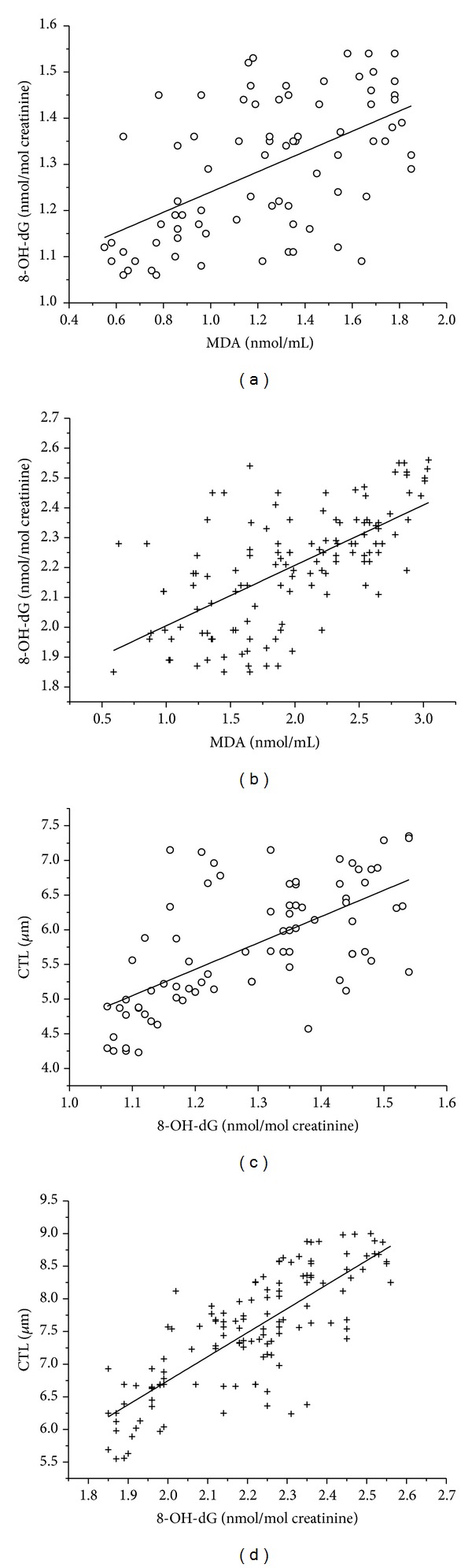
Correlation between MDA level and 8-OH-dG level in lymphocytes of workers in control group ((a), *r* = 0.556, *P* < 0.001) and exposed group ((b), *r* = 0.640, *P* < 0.001), and correlation between 8-OH-dG and CTL level in lymphocytes of workers in control group ((c), *r* = 0.644, *P* < 0.001) and exposed group ((d), *r* = 0.794, *P* < 0.001).

**Figure 2:**
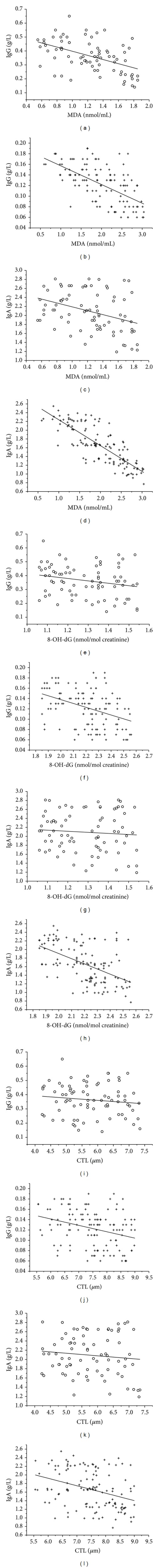
Correlation between MDA and IgG, IgA level in lymphocytes of workers in control group ((a), *r* = −0.498, *P* < 0.001; (c), *r* = −0.363, *P* = 0.001) and exposed group ((b), *r* = −0.606, *P* < 0.001; (d), *r* = −0.814, *P* < 0.001), respectively; correlation between 8-OH-dG and IgG, IgA level in lymphocytes of workers in control group ((e), *r* = −0.229, *P* = 0.004; (g), *r* = −0.080, *P* = 0.486) and exposed group ((f), *r* = −0.405, *P* < 0.001; (h), *r* = −0.523, *P* < 0.001), respectively; correlation between CTL and IgG, IgA level in lymphocytes of workers in control group ((i), *r* = −0.126, *P* = 0.270; (k), *r* = −0.110, *P* = 0.339) and exposed group ((j), *r* = −0.312, *P* < 0.001; (l), *r* = −0.350, *P* < 0.001), respectively.

We observed a significant correlation in MDA and 8-OH-dG levels, 8-OH-dG, and CTL levels in the control group (*r* = 0.556, *P* < 0.001; *r* = 0.644, *P* < 0.001; resp.) (Figures 1(a) and 1(c)) and in the exposed group (*r* = 0.640, *P* < 0.001; *r* = 0.794, *P* < 0.001; resp.) (Figures 1(b) and 1(d)). Also, we examined the correlations in genotoxic/oxidative damage biomarkers and immunoglobins of exposed workers and control workers next to further evaluate a possible role and a potent biomedical significance of immunoglobin levels in genotoxic/oxidative damage. The correlation between MDA, 8-OH-dG, CTL, and IgG, IgA of workers exposed to coke oven emission is plotted in Figures 2(b), 2(d), 2(f), 2(h), 2(j), and 2(l). As can be seen, there is a significant negative correlation between MDA, 8-OH-dG, CTL, and IgG, IgA levels in exposed workers (*P* < 0.001). A similar significant negative correlation between MDA and IgG, IgA level, 8-OH-dG, and IgG in control workers (Figure 2(a), *r* = −0.498, *P* < 0.001; Figure 2(c), *r* = −0.363, *P* = 0.001; Figure 2(e), *r* = −0.229, *P* = 0.004, resp.) was observed. However, in the control group, 8-OH-dG levels were not significantly correlated with IgA levels (Figure 2(g), *r* = −0.080, *P* = 0.486). Also, CTL levels were not significantly correlated with IgG and IgA in control workers (Figure 2(i), *r* = −0.126, *P* = 0.270; Figure 2(k), *r* = −0.110, *P* = 0.339, resp.). Further, PAH exposure enhances this negative correlation. Interestingly, although most individuals with low IgG, IgA levels tended to have more MDA, 8-OH-dG, and CTL levels, some with high IgG, IgA also had more MDA, 8-OH-dG, and CTL levels as can be seen particularly in Figures 2(b), 2(f), 2(h), 2(j), and 2(l), respectively.

## 4. Discussion

The working environment at a coke plant can negatively affect the employed workers who are exposed to coke oven emissions containing PAHs [24]. PAHs are introduced to the environment in numerous ways and are ubiquitously distributed. They are taken up by humans at the workplace, by smoking, by breathing polluted air, and by consuming food and medicines. They are potent oxidative/genotoxic damage and immunotoxic compounds. Current occupational exposure to PAHs was investigated in China coke oven plants in order to establish which resulted in oxidative/genotoxic DNA damages and immunotoxic effects which are considered to be indicators for long term adverse health effects. Biological monitoring of the internal dose was based on the analysis of urinary 1-hydroxypyrene. Biological effect monitoring including MDA, 8-OH-dG, CTL, CTM, MN, CA and IgA, IgE, IgG, and IgM levels as well as the correlation among these markers were evaluated.

In the present study, the coke oven workers seemed to have been exposed to a significant high level of PAHs based on the urinary 1-OHP. The PAH exposure of coke oven workers in the present study was similar with that in Siwińska et al.'s [16] and in Zhang et al.'s [25] study conducted in coke oven workers and much higher than that reported by Pavanello et al. [26] in CMBN assay in coke oven workers. Though the length of exposure is not, therefore, the only risk factor, Yang et al. reported that the length of time spent outdoors had marginal positive associations with urinary 1-OHP levels in nonoccupationally exposed Koreans [27]. So, significantly higher length of employment time may promote a synergistic effect on excretion of 1-OHP in PAH exposed coke oven workers. Significant distributions for alcohol users and no significant distributions for smoking status in exposed coke oven workers were observed in the present study. On the contrary, no significant difference was found in alcohol users between control and coke oven workers in Leng et al.'s [28] and Zhang et al.'s studies [25]. Other studies [29, 30] indicated that the smoking of cigarettes significantly increases the urinary 1-OHP concentration, while alcohol consumption does not affect the urinary 1-OHP concentration. We further analyzed the smokers and nonsmokers in control and exposed group. The findings indicated the smoking was actually increase 1-OHP levels but not significance. This may be partially due to the relatively higher exposure to PAHs, which may hide the effect of smoking on 1-OHP concentrations [25]. Hence, the smoking life style may not significantly affect the urinary 1-OHP concentration in this study.

Metabolic transformation of PAHs generates reactive electrophilic metabolites, causing DNA damage and triggering the production of reactive oxygen species that lead to oxidative stress [31]. The analysis of urinary excretion of 8-OH-dG is a useful approach to assess individual cancer risk due to oxidative stress [32]. Data from the present study also showed that levels of MDA, 8-OHdG was associated with exposure to carcinogenic PAHs among coke oven workers. Significant differences were observed for CTL levels, MN, and CA frequency of PAH exposed workers. In agreement with our results, Siwińska et al. reported that the level of urinary 1-OHP was correlated with genotoxic effects in coke oven workers as determined by a number of assays, including micronuclei frequency and DNA damage measured by sister-chromatid exchange and comet assays [16]. Significant correlation in MDA and 8-OH-dG levels, 8-OH-dG, and CTL levels in all subjects was observed in the present study. It is well known that reactive oxygen species generated by PAHs may induce damage to DNA. It is implied that oxidative DNA modification and oxidative DNA damage generated by PAHs exposed in coke oven workers [33]. High levels of MDA, 8-OHdG, and DNA damage were also indicated by several occupational studies [3, 8, 34]. Additionally, accumulating evidence suggested that ethanol can induce oxidative DNA damage in humans [3, 35]. The significant distributions for alcohol users in exposed coke oven workers indicates that alcohol drinking may synergetically increase levels of MDA, 8-OHdG, and DNA damage in alcohol users of coke oven workers.

A general observation has been that the immune system of humans is the most sensitive to modulation by PAHs. Four kinds of immunoglobulins were lower in coke oven workers, but this was significant only for IgG and IgA (*P* < 0.001). The results of this study regarding immunoglobulin levels also show consistent with the data of Szczeklik et al. [36] who demonstrated biosynthesis of IgG and IgA was markedly depressed immunoglobulins in serum of coke oven workers. Oh et al. [13] found that the four kinds of immunoglobulin types were lower in automobile emission inspectors, but this was significant only for IgG (*P* = 0.047). On the other hand, Winker et al. [37] did not find any alterations of the serum immunoglobulin levels in PAH-exposed workers. Similarly, the four kinds of immunoglobulin types were found in lower amounts in the waste incineration workers, but this disparity was not significant one [38]. By contrast, there was also a significant enhancement in serum IgG levels and the percentage of monocytes in the PAH exposed asphalt workers compared to the control group [12]. In addition, Szczeklik et al. also found that serum IgE had a trend toward increased values in serum of coke oven workers [36]. These may be due to the fact that the place of adaptive responses or changes may come to plays in situation of chronic exposure [39]. Generally, IgG plays a major role in humoral immune response through complement fixation and neutralization, and reduction in IgG may lead to downregulation of antibody-mediated host resistance, which could enhance influence of infection or tumor progression [40, 41]. The decrease in IgA production suggests that PAHs may compromise protection of mucosal surfaces in the respiratory tract [42]. There has been reported that many PAHs are known to be potent suppressors of the immune response. Additionally, the immunosuppressive potential of PAHs is linked to their carcinogenic potency [43]. Accordingly, in some extent, the PAH exposure in coke oven workers showed the immune depression and the increase in potential carcinogenic effect through the significant reduction of IgG and IgA levels. Further, except age, alcohol status, smoking status, IgM, IgE, and other markers were significantly associated with PAH exposure as indicated by 1-OHP levels. As previous descriptions, the markers of employment time, MDA, 8-OH-dG, CTL, CTM, MN (‰), CA, IgG, and IgA may be more important markers in coke oven workers.

Further, a significant negative correlation in genotoxic/oxidative damage biomarkers of MDA, 8-OH-dG, CTL levels, and immunoglobins of IgG, IgA levels, especially in coke oven workers, was found in the present study. Studies have suggested that oxidative stress-induced lipid peroxidation may play a role in toxic responses to PAH in immunological systems. For example, Zhigacheva et al. found that the increased lipid peroxidation products were linked to modification of leucocyte membranes and reduced activity of lymphocyte enzymes in animals after 6 months of PAH inhalation as compared with controls [44]. In addition, moderate activation of lipid peroxidation and oxidative damage as determined by plasma MDA and 8-OHdG levels were detected simultaneously with significant alterations in IgA and IgE levels described by Jeng et al. [42]. Also, a significant difference was found regarding the levels of IgE and the levels of DNA damage in polynuclear cells were found in waste incineration workers described by Oh et al. [38]. Further, PAH exposure enhances this negative correlation (Figure 2). Generally speaking, PAHs are cytotoxic at high doses, while lower doses of PAHs frequently result in alteration of immunological responses. It is likely that heavier PAH species increased redox activity and subsequently induced generation of ROS [42] which altered IgE and IgA levels in workers exposed to PAHs. Further research should clarify the effects of high molecular weights of PAHs on immunological function.

The study also has some limitations. First, our sample size is relatively small and this is one of the most common reasons for the failure to replicate reported associations across studies [45]. This may be due to no significance for 1-OHP levels in smokers and nonsmokers, IgM, IgE levels between the control and exposed group. In particular, the number of subjects in CA assay was only 10 for control and 36 in exposed group. So, further investigation still recommends large sample population in the future. On the other hand, though alcohol consumption does not affect the urinary 1-OHP concentration [29, 30], excess alcohol consumption may result in the enhancement of PAH metabolites [25] and the mechanism is needed further study. Finally, staff members of the offices and hospitals of the same steel company served as the control group. There may be differences in economics and nutrition status between control and coke oven workers. This may be affected by food intakes which are also known as an important route of exposure to PAHs [9]. To better understand the factors related to the increase of 1-OHP, the food consumption, social activities, and nutrition status should be included in the questionnaire and further analyzed in the future.

## 5. Conclusion

In conclusion, the present study provided yet more evidence that PAHs cause significant oxidative stress/DNA damage through the MDA, 8-OHdG levels and CTL level, CTM, MN, and CA frequency in coke oven workers. Moreover, our immunoglobulin study showed that IgG and IgA were significantly reduced by PAHs. In addition, our investigation of the association between MDA and 8-OH-dG levels, 8-OH-dG and CTL levels, MDA, 8-OH-dG, CTL levels, and immunoglobins of IgG, IgA levels was proven to be a meaningful or relevant association. It is implied that oxidative stress/DNA damage induced by PAHs may play a role in toxic responses for PAH in immunological systems.

## Figures and Tables

**Table 1:** Demographic characteristics of workers in the control and exposed groups.

Variables	Control group (78)	Exposed group (126)	*P* value
1-OHP (*μ*mol/mol creatine)	0.55 ± 0.28	9.36 ± 2.14∗∗∗	<0.001^a^
Age (year)	32.4 ± 4.9	33.6 ± 5.2	0.103^a^
Employment time (year)	12.7 ± 2.5	14.1 ± 2.7∗∗∗	<0.001^a^
Current smokers, yes (%)	41 (52.6%)	79 (62.7%)	0.153^b^
Alcohol users, yes (%)	18 (23.1%)	57 (45.2%)	0.001^b^
MDA (nmol/mL)	1.23 ± 0.37	1.97 ± 0.61∗∗∗	<0.001^a^
8-OH-dG (nmol/mol creatinine)	1.29 ± 0.15	2.20 ± 0.19∗∗∗	<0.001^a^
CTL (*μ*m)	3.16 ± 0.58	7.49 ± 0.89∗∗∗	<0.001^a^
CTM	0.85 ± 0.23	10.48 ± 3.52∗∗∗	<0.001^a^
MN (‰)	1.83 ± 0.51	2.78 ± 0.63∗∗∗	<0.001^a^
CA (N, AR%)	10, 12.8%	36, 28.6%	0.009^b^
IgG (g/L)	0.36 ± 0.11	0.12 ± 0.03∗∗∗	<0.001^a^
IgA (g/L)	2.09 ± 0.44	1.66 ± 0.44∗∗∗	<0.001^a^
IgM (g/L)	1.76 ± 0.69	1.62 ± 0.72	0.172^a^
IgE (IU/mL)	173.90 ± 30.24	167.76 ± 43.16	0.273^a^

1-OHP: 1-hydroxypyrene; CTL: comet tail length; CTM: comet tail moment; MN: micronucleus; CA: chromosomal aberrations. Values are shown mean ± SD except where indicated. ^a^when compared with control by Mann-Whitney test, ^b^when compared with control by chi-square test.

***P < 0.001.

**Table 2:** 1-OHP levels by smoking use status in control and exposed group.

	Control group		Exposed group	
	Smoking (No)	Smoking (yes)	Smoking (No)	Smoking (yes)
*n*	37	41	47	79
1-OHP level	0.48 ± 0.21	0.57 ± 0.31	8.62 ± 2.12	9.58 ± 3.65
*P* value^a^	0.142		0.096	

^a^when compared between nonsmoking and smoking users control and exposed group by chi-square test.

**Table 3:** Dose-response relationship with the levels of 1-OHP with immunoglobins.

1-OHP level	IgG (g/L)	IgA (g/L)	IgM (g/L)	IgE (IU/mL)
	*β*	*β*	*β*	*β*
	(95% CI)	(95% CI)	(95% CI)	(95% CI)
	*P*	*P*	*P*	*P*
Control group	−0.109	−0.472	−0.051	−0.067
	(−0.359–0.130)	(−0.438–0.296)	(−0.445–0.283)	(−0.941–0.518)
	0.353	<0.001	0.658	0.565

Exposed group	−0.312	−0.538	−0.062	−0.071
	(−0.3455–0.176)	(−0.463–0.211)	(−0.903–0.465)	(−0.233–0.098)
	<0.001	<0.001	0.592	0.420

**Table 4:** Correlations of 1-OHP levels and the other studied variables in total subjects.

Variables	*r* ^a^	*P*-value^b^
Age (year)	0.157	0.233^a^
Employment time (year)	0.455	<0.001^a^
Current smokers, yes (%)	0.329	0.073^b^
Alcohol users, yes (%)	0.165	0.209^b^
MDA (nmol/mL)	0.623	<0.001^a^
8-OH-dG (nmol/mol creatinine)	0.668	<0.001^a^
CTL (*μ*m)	0.571	<0.001^a^
CTM	0.682	<0.001^a^
MN (‰)	0.459	<0.001^a^
CA (N, AR%)	0.422	0.009^b^
IgG (g/L)	−0.732	<0.001^a^
IgA (g/L)	−0.677	<0.001^a^
IgM (g/L)	−0.209	0.127^a^
IgE (IU/mL)	−0.187	0.211^a^

^a^Spearman rank correlation coefficient. ^b^Spearman rank correlation coefficient for the comparisons between each of the studied variables.
